# Perceived needs of health tutors in rural and urban health training institutions in Ghana: Implications for health sector staff internal migration control

**DOI:** 10.1371/journal.pone.0185748

**Published:** 2017-10-05

**Authors:** Robert Kaba Alhassan, Christopher B. Beyere, Edward Nketiah-Amponsah, Prudence P. Mwini-Nyaledzigbor

**Affiliations:** 1 School of Nursing and Midwifery, University of Health and Allied Sciences, Ho, Ghana; 2 Health Training Institutions Unit, Ministry of Health Headquarters, Human Resources for Health Directorate, Accra, Ghana; 3 Department of Economics, University of Ghana Legon, Accra Ghana; B P koirala Institute of Health Sciences, NEPAL

## Abstract

**Background:**

The population of Ghana is increasingly becoming urbanized with about 70% of the estimated 27 million people living in urban and peri-urban areas. Nonetheless, eight out of the ten regions in Ghana remain predominantly rural where only 32% of the national health sector workforce works. Moreover, the rural-urban disparities in the density of health tutors (staff responsible for pre-service training of health professionals) are enormous. This paper explores perceived needs of health tutors in rural and urban health training institutions in Ghana.

**Methods:**

This is a descriptive qualitative study conducted in the Greater Accra and Northern regions of Ghana. The Study used the deductive thematic and sub-thematic analysis approaches. Five health training institutions were randomly sampled, and 72 tutors engaged in separate focus group discussions with an average size of 14 participants per group in each training institution.

**Results:**

Perceived rural-urban disparities among health tutors were found in the payment of extra duty allowances; school infrastructure including libraries and internet connectivity; staff accommodation; and opportunities for scholarships and higher education. Health tutors in rural areas generally expressed more frustration with these work conditions than those in urban areas.

**Conclusions:**

There is the need to initiate and sustain work incentives that promote motivation of rural health tutors to control ongoing rural-urban migration of qualified staff. It is recommended the following incentives be prioritized to promote retention of qualified health tutors in rural health training schools: payment of research, book and rural allowances; early promotion of rural staff; prioritizing rural tutors for scholarships, and introduction of national best health tutor awards.

## Introduction

The 2006 World Health Organization (WHO) report stated that “developing capable, motivated and supported health workers is essential for overcoming bottlenecks to achieve national and global health goals” [1]. This statement elucidates the critical role health workforce play in the realisation of health goals globally.

The health service industry, like other service industries, is labour-intensive and service providers represent (to a large extent) the core values of the industry in every country [1]. This is why pre-service training for health workers in health training institutions must be given the needed attention. Pre-service training for health workers is particularly important in developing countries such as Ghana which is still grappling with limited numbers of motivated healthcare workers to meet the growing population health needs.

Even though policy makers have acknowledged some health sector human resource challenges and in some cases have initiated remedies, the impact of such interventions appear to be skewed and insufficient in addressing the existing challenges [2–5]. Over the years, emphasis on health worker motivation has often been limited to clinical health staff working in hospitals and other health care facilities [2,3,6,7]. Consequently, health tutors who are largely responsible for the training and development of skills and knowledge of clinical health staff appear to have been receiving lesser attention in health worker motivation strategies and initiatives.

Moreover, health tutors, particularly in nursing and allied health training schools, have not been adequately engaged in many previous studies on health worker motivation and retention. As a result, “wholesale” incentive packages are often rolled out for all categories of health workers in Ghana. Albeit the Ministry of Health (MoH) recognizes health tutors as important input factors in the production of high quality health professionals for the health sector [8], there appears to be limited empirical data on factors that influence motivation and retention of health tutors in Ghana.

It is frightening that Health tutors play an essential role in sustaining health training institutions in Ghana yet little is known of the working conditions of this cadre of health workers and the perceived rural-urban disparities in work incentives. In light of this, the MoH is confronted with the increasing negative effects of rural-urban migration of qualified health tutors for better working conditions in the more endowed regions of the country [8]. In Ghana, the health tutor-student ratio in a typical urban health training institution is 1:80 compared to 1:150 in a typical rural training institution [8].

The impact of rural-urban imbalance on quality teaching and learning is not farfetched. Health trainees without the requisite pre-service training are potential threats to clients and the healthcare system at large. For instance, students are likely to receive poor training from demotivated health tutors which could compromise the quality of services they eventually render to clients.

Since quality of health services remains a major concern to clients, policy makers and stakeholders of public health in Ghana [6,7,9,10], a comprehensive staff motivation approach to health care quality improvement is imperative, particularly the need to focus on health tutors since they are critical in ensuring quality pre-service training for health professionals.

Overall, this paper is motivated by gaps in the literature on health tutor motivation, and the implications on quality pre-service training of health professionals. This descriptive qualitative study explored the perspectives of health tutors on factors that determine their motivation and retention in public health training institutions regulated by the MoH in Ghana. Understanding perspectives and expectations of health tutors will help inform policy reforms towards improving health tutor motivation and retention. This paper measured views of health tutors on work conditions and the perceived rural-urban disparities in work incentives.

## Methods

### Study design and sampling strategy

This is a descriptive qualitative study conducted in the Greater Accra (GAR) and Northern regions (NR) of Ghana, using the deductive thematic and sub-thematic analysis approach. These two regions were purposively selected for rural–urban balance since the GAR has relatively more health training institutions located in urban areas while health training institutions in the NR are predominantly located in rural areas. This design was adopted because there was the need to identify basic social processes related to health tutor motivation behaviours in the training institutions where they work.

A total of six health training institutions were purposively selected and earmarked for Focus Group Discussions (FGDs) based on maximum variation sampling taking into consideration the different characteristics of the training institutions in the two region.

The health training institutions were selected based on their individual characteristics– student population, number of health tutors, and the age of the training institutions. Age was considered to ensure a balance between old and relatively young training institutions. In all, five out of the six selected training institutions participated in the focus group discussions, involving 72 health tutors. Nonetheless, saturation was attained after conducting the five focus group discussions. The average number of participants per focus group discussion (FGD) was 14 and one trained moderator.

The selection of health tutors within the individual health institutions was mainly purposive, since the focus was to explore experiences of health tutors who have worked for not less than six months and were posted by the Ministry of Health Human Resource Directorate (HRD) to the health institutions. Based on this criteria, all 72 health tutors who qualified and were approached agreed to participate in the study. Hence the response rate was 100%. The study was conducted in direct collaboration with the Ministry of Health headquarters (the Health Training Institutions Secretariat at that time), and principals of the various training institutions.

### Data collection procedure

Piloting of the focus group discussion guide was done on 19^th^ April, 2016 in two conveniently sampled health training institutions in the Eastern Region of Ghana (1 Rural and 1 Urban) to correct possible ambiguities and typographical errors prior to full scale utilization of the tool on the field. The main focus group discussions were conducted from 21^st^ April to 14^th^ July, 2016. Each focus group discussion lasted approximately 55 minutes. One lead researcher, two trained research assistants, one driver and one vehicle were involved in the data collection. Logistics used included two audio recorders, 80 copies of focus group discussion guides, pens, pencils, erasers, notebooks, tape recorders and name tags.

### Data collection instruments

Data collection instruments were basically focus group discussion guides which were structured in five main sections, namely: Introduction; Section A (Present Job and Perspectives on Workplace Motivation Factors); Section B (Perceived disparities in health tutor motivation); Section C (Staff and Student Situation in Training Schools); Section D (Institutional and Staff Performance Targets). These sections formed the thematic areas for the focus group discussions and were informed by reviewed literature on workplace motivation and also responses from the pilot interviews. Most questions were structured in open-ended fashion to elicit opinions of health tutors on factors that motivate and constrain them. See details of qualitative data collection tool in the attached supporting information file [S1 File].

### Consent to participate

In line with the ethics protocols of research in Ghana, written informed consent was signed by all focus group discussion participations prior to the discussions. The researchers also obtained written permissions from the Ministry of Health headquarters, Health Training Institutions Unit (at the time) and also from principals of all training institutions involved.

### Operational definitions

Health tutors: For the purposes of this study, health tutors include professional nurses, midwives, public health nurses and other allied health professionals who have been appointed to teach in MoH regulated health training institutions.

Health training institutions: Health training institutions in the Ghanaian context are tertiary institutions either set out to train only nurses, nurses and midwives, health assistants (clinical), health assistants (preventive) or allied health professionals in the disciplines of disease control, community nutrition, health information etc. The focus of this study was on health training institutions that train general nurses, midwives and health assistants (clinical).

### Data management and analysis

The focus group discussions and key informant interviews were recorded and later manually transcribed verbatim. The researcher then develop four key thematic areas based on the transcripts. Sub-themes were developed following content analysis which was based on similarities in content of responses. Moreover, a review of existing literature on worker motivation theories informed the development of the thematic areas. Qualitative analysis was done by three of the four authors of this paper. The field notes complemented the content analysis, and development of sub-themes from the responses. The four key thematic areas are:

- Perceived motivation factors and constraints
- Perceived disparities in work conditions of health tutors
- Perceived push and pull factors for staff internal and external migration
- Recommendations from focus group participants on motivation levels and staff retention

Descriptive statistics were presented on staff demographics and background information on the sampled health training institutions. Researchers used administrative records, with the permission from management of the institutions, for information on respondents’ gender, age, educational qualification and other characteristics. This was done prior to the focus group discussions.

### Credibility and internal validity of findings

Co-authors double checked the responses for their truthfulness or otherwise and so credibility of findings of the study is assured. Moreover, the researchers did cross referencing of research subjects’ responses to identify contradictions. Where there were contradictory instances, an independent validator was employed to do further content analysis to isolate untruths and misrepresentation of facts. Also, the researchers triangulated the data collection procedures such as focus group discussions, secondary data and individual interviews to look for possible divergence and convergence of patterns.

### Auditability and confirmability

To promote auditability of the responses, the social, physical and interpersonal contexts associated with the data were ensured by recording the gender, work history, age and other personal/work related information of the respondents. Moreover, all the authors did a theoretical coding thereby enabling a minimization of potential bias and coding error rates.

### Fittingness and transferability

Analytical generalizability of the findings could be a challenge in this study because the study was conducted in two out of ten regions of Ghana. It is possible the responses were peculiar to the work conditions of the health training institutions in the two regions and might not necessarily be a true reflection of the national picture. Nonetheless, selection of the Greater Accra (predominantly urban) and Northern regions (predominantly rural) gives a fair representation of the country considering that the main objective of the study is a rural-urban comparison of workplace motivational factors among health tutors.

### Findings

#### Training schools involved in focus group discussions

From the descriptive data, the average age of health tutors was 42 years in GAR and 34 years in NR. In both regions, it was found, the youngest age quoted by respondents was approximately 30 years and oldest quoted age was 59 years. Close to 60% of health tutors engaged in the NR were not permanent workers while the remaining were permanent staff on government of Ghana (GoG) payroll. In the GAR, over 70% of health tutors who participated in the focus group discussions were full time or permanent health tutors on GoG payroll and fewer than 30% were part time staff.

In terms of gender distribution, out of the 72 participants, the ratio of female to male tutors was higher for females in GAR (approximately 4:1) compared to a lower ratio for females in NR (approximately 1:3).

Moreover, it was observed that more health tutors in GAR had higher educational qualification (minimum of Masters Degree) than tutors in NR, who predominantly had a maximum of Bachelors Degree. About 60% of health tutors in GAR had Masters Degree; less than 10% of health tutors in NR had Masters Degree.

In terms of tutor: student ratio, *GAR School B* had the highest of 1:70 while *GAR School A* had the lowest of 1:33. *NR School A* recorded the highest student population of approximately 1,300 as at 2015 while *NR School C* recorded the lowest student population of 494. Total student populations for *NR School A* and *NR School B* were approximated based on average student intake for the periods of 2011–2015, see details in Table 1.

**Table 1:** Health tutor density in selected schools.

Institution	Tutor: student ratio	No. Students	No. Tutors
NR School A*^β^	1:54	1,300	24
NR School B* ^β^	1:69	1,233	18
NR School C^∞^	1:38	494	13
GAR School A^β^	1:33	953	29
GAR School B^β^	1:70	1,259	18
GAR School C^§^^+^	NA	NA	NA

Legend:

*Figures are approximations based on previous school records

^β^Nursing and Midwifery Training College

^∞^Nursing Training College

^§^Midwifery and Health Assistant (Clinical) school

^+^School did not participate in FGDs; NA (Not available)

### Qualitative findings

#### Perceived workplace motivation factors

During the focus group discussions, health tutors were asked to express their views on incentives at their workplace. From the focus group discussions, health tutors generally perceived a well-motivated staff to be one who is appreciated by school management/administration. According to participants of the focus group discussion, management could show appreciation by giving money or fuel allowances to tutors whenever they perform extra duties. A tutor from one of the schools in the GAR lamented:

*“…I have a problem with [school] administration*. *When I make rounds [performs a duty] for the school…I don’t get any support in the form of fuel and out of station allowances…if even we are paid the amount is so small…”* [Urban FGD Female participant].

Some participants however appeared to be motivated by the good working relationship they have with their school management. A participant from the NR, indicated that the cordial working relationship with school administration, particularly the principal was his major source of motivation:

*“*…*to me the relationship [with school management] is cordial…our principal listens to concerns of tutors and willing to take on board our suggestions…”* [Rural Male participant].

A similar view expressed by a participant suggested the kind of work relationship health tutors have with their principals could either motivate or demotivate staff at the workplace.

*“…personally I also have good working relationship with the school’s administration…though we sometimes have disagreements…generally am ok with the relationship with administration…”* [Rural FGD Male participant].

Participants of the Focus group discussion in the GAR emphasized monetary incentives as a way of motivating health tutors. GAR participants argued that giving additional financial incentives for extra work done is one way to improve motivation levels for health tutors. Discussions from the GAR focus group discussions generally revealed that many tutors were not satisfied with existing financial incentive packages for tutors. This is evident in the contribution made by a participants during the focus group discussion.

*“*…*in terms of financial incentives…I think tutors should be paid allowances for doing additional work…”* [Urban FGD Female participant].

During the focus group discussion in *GAR School B*, some specific financial incentives were identified as key in increasing motivation levels of health tutors, and in improving the quality of teaching and learning in health training institutions.

*“*…*these [financial] allowances are fuel*, *vehicle maintenance*, *rent*, *research and book allowances…”* [Urban FGD Female participant].

Another form of motivational package that dominated the focus group discussions in GAR and NR was the need to re-introduce the system that allow health workers including health tutors acquire vehicles through hire purchase. They reckoned this will help reduce the burden of purchasing vehicles from the open market. A tutor from *GAR School A* contributed to the discussion indicating that:

*“*…*the hired purchase cars should be reinstated to motivate health tutors…buying from the open market is too expensive for the average health tutor to acquire…”* [Urban FGD Male participant].

Focus group discussions in the GAR particularly revealed that health tutors were not happy with the current retirement plan for health tutors where they largely depend on the Social Security and National Health Insurance Trust (SSNIT). Participants thus, suggested the need for government to institute better retirement plans for health tutors. One participant intimated:

*“…better retirement plans should also be instituted for health tutors like other sectors to guarantee decent retirement*…*”* [Urban FGD Male participant].

Opportunity for early promotions appeared to be of more concern to health tutors in the NR than their counterparts in the GAR. Health tutors in the NR perceived their promotions were delayed more than their counterparts in better endowed regions in Ghana such as the GAR. For instance, a health tutor in *NR School expressed* her frustrations stating:

*“*…*another problem I have is delayed promotion for some of us [Health Tutors]…even though I completed school with some people they [colleague tutors elsewhere] are currently my seniors because they were promoted before me…”* [Rural FGD Female participant, 2016].

Another participant corroborated saying: *“…I was due for promotion in my former post before coming here but when I came here I was told to wait because I wasn’t a staff here…which is unfair…”* [Rural FGD Male participant].

Even though there was no proof of the participants’ allegations, other members of the group supported these stories; responses from other FGD members suggest perhaps some health tutors were not promoted when they were due for promotion. The reasons for this were not immediately confirmed in this study.

Many participants also expressed dissatisfaction with the job description for health tutors. Health tutors generally believed their job descriptions were either inadequately documented or not documented at all in some cases. Moreover, management kept changing job descriptions of health tutors without official notification or directive from the employer, the Ministry of Health (MoH). One participant lamented as follows:

“*…Job description of tutors not revised regularly in consultation with tutors…*.*though our roles and responsibilities keep changing on paper*, *we use the same job descriptions…”* [Rural FGD Male participant

Likewise, health tutors intimated the need to have conditions of service as is the case for other health professionals:

*“*…*conditions of service should come with the job description*....*we [health tutors] don’t have conditions of service…*.*and [thus] should be provided with conditions of service…”* [Rural FGD Male participant].

Participants also emphasized opportunities to pursue higher education as a very important motivational factor. However, some tutors perceived that there is inequity in accessing available opportunities; some tutors in endowed regions have better advantage over tutors working in the rural areas. A tutor from *NR School C* focus group discussion stated that:

*“…I will suggest health tutors are sponsored to pursue their higher education through exchange programmes besides increasing salaries…”* [Rural FGD Male participant].

*“…I also think these opportunities are currently limited…one only gets to hear of them after the people [beneficiaries] have return…we don’t usually hear when the adverts are placed and the criteria used to select the people…”* [Urban FGD Female participant].

Other workplace motivational factors identified by participants in the focus group discussions are conducive work environment (especially office space); active involvement of tutors in school activities; improve availability of teaching and learning materials; availability of reliable internet connectivity; descent accommodation for health tutors, and provision of means of transportation to work.

*“…We don’t have tutors’ offices…we share the same office which also serves other purposes…We [the school] don’t also have enough teaching and learning aides…laptops…computers…anatomical dummies…”* [Rural FGD Male participant].

**Fig 1:**
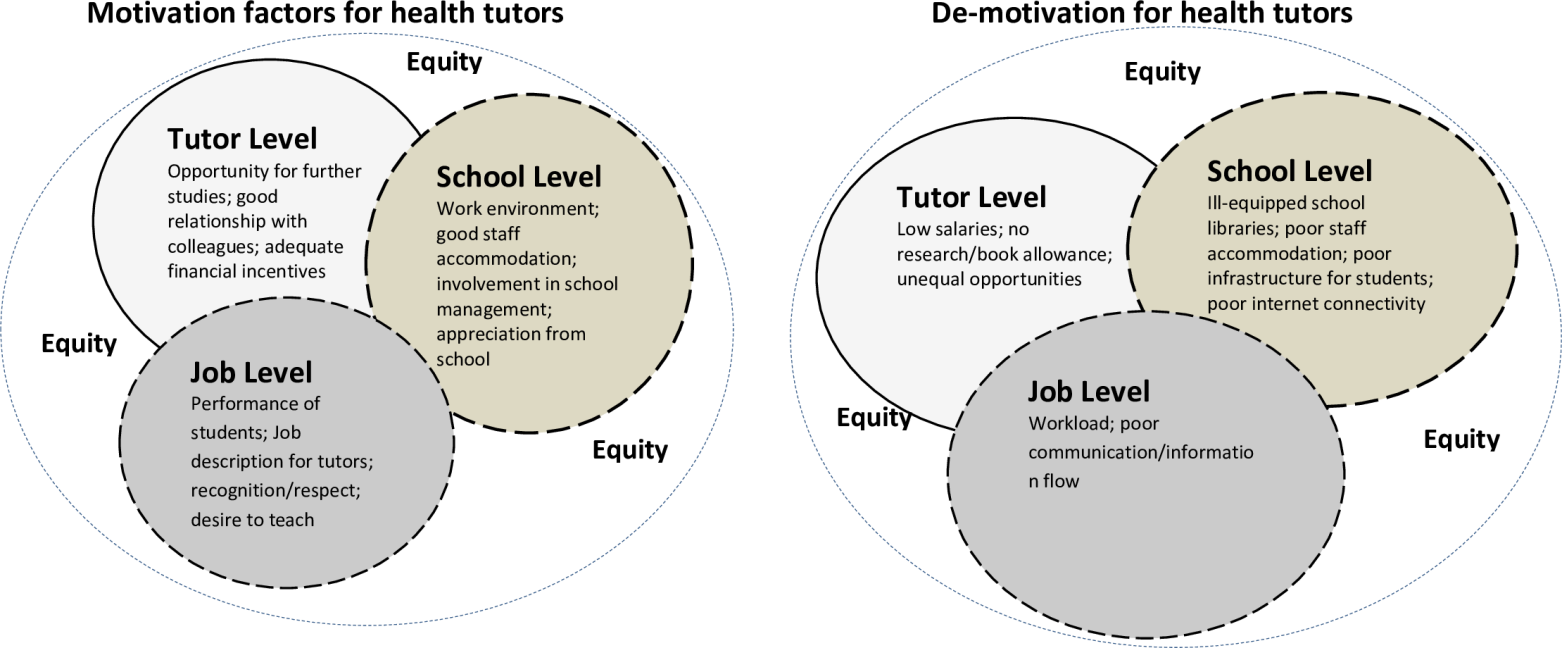
Motivation and de-motivation factors for health tutors. A (Motivational factors); B (De-motivational factors); Source: Conceptualized by authors (2017).

Some participants said they were motivated mostly by the good interpersonal relationship with colleague tutors; the good performance of students in professional examinations, and seeing past students at the clinical sites practising as nurses. Fig 1 illustrate the key themes that emanated from the focus group discussions in the GAR and NR.

*“…Students performing well in their final examinations [NMC licensing examinations] …it is a sign that the tuition went well and that they [students] understood everything…”* [Urban FGD Female participant].

### Perceived workplace constraints/disincentives

Responses from participants also suggested health tutors were demotivated in their workplaces because of constraints such as poor internal communication in schools; lack of clear organogram and chain of command (especially in rural schools); undefined and/or conflicting roles for tutors; multiple tasking without commensurate motivation for health tutors; large class size coupled with low tutor population; perceived power struggle among tutors, and longer travel time to work (especially for urban tutors).

*“*…*I have to travel…to school over several hours*…*I get exhausted by the time I get to school*…*”* [Urban FGD Male participant].

*“…I for instance also double as the academic tutor and will sometimes get home after 9pm…even though we don’t get paid for the extra work done…*..*we are doing this sacrificial work for…mother Ghana…”* [Rural FGD Male participant].

*“…The work load is huge but we don’t get extra work allowance to motivate us…”* [Rural FGD Female participant].

### Perceived disparities in work conditions of health tutors

Further content analysis of the focus group discussions showed that the response patterns of rural and urban health tutors varied. For instance, it was found that tutors in urban schools mentioned more of financial incentives and means of transport to work as key sources of motivation while rural tutors were particular about opportunity for higher education and full employment (for casual workers) by the MoH.

Another variation in the tutors’ responses was that whereas tutors in urban areas complained of the lack of accommodation facilities for tutors, tutors in rural facilities decried poor school infrastructure and inadequate teaching/learning materials. Moreover, challenges with internet access and its consistency were predominantly mentioned during discussions in rural schools than those in urban schools.

It was also found that health tutors in rural schools were constrained by lack of career development opportunities and ill-equipped libraries. Tutors in urban schools were rather constrained by longer travel time to work. Furthermore, lack of organized administrative structures in pertinent schools was more associated with rural than urban schools, according to responses from the focus group discussions.

Responses of the health tutors in the various focus group discussion revealed that, tutors generally perceived unfairness and unequal access to work incentives especially among the rural and urban tutors.

*“…I think these things [inequities in staff motivation] bring internal conflicts and misunderstandings…people feel cheated and don’t put up their best…”* [Urban FGD Female participant].

Moreover, it was noticed that within schools, some tutors got better favours from school heads and management than others. Some tutors expressed dissatisfaction with unequal treatment for tutors, access to information and other career opportunities.

*“*…*I don’t know about other tutors…but for me I think the head sometimes takes decisions that appear to favour some tutors…I don’t want to talk too much but what I know is that there is the need to have national policies on what should be done across board for all health tutors…this is my personal opinion…”* [Urban FGD Male participant].

*“…in this school sometimes I feel some tutors are treated better than others…if you ask some of my colleagues here they will tell what am saying…”* [Urban FGD Female participant]

*“…this differential treatment creates bitterness and divisions among the tutors and this alone can result in demotivation because of disunity…we need to support each other but when there is disunity…this cannot happen…”* [Rural FGD Male participant].

### Perceived push and pull factors for staff internal and external migration

Participants of the various focus group discussions mentioned factors that attracted them (pull factors) to teach as health tutors in their current workplace. The pull factors that recurred throughout the discussions include the following: love for the work (i.e. intrinsic desire to teach); willingness to help reform health trainees before practice at the ward; desire to work with particular principal of the school; job security since (it is government employment); low cost of living in area where school is located; better opportunity for career upgrading; perceived unity/team work among tutors in school; proximity to family; anticipated support from school for higher education; respectful and disciplined students.

**Fig 2:**
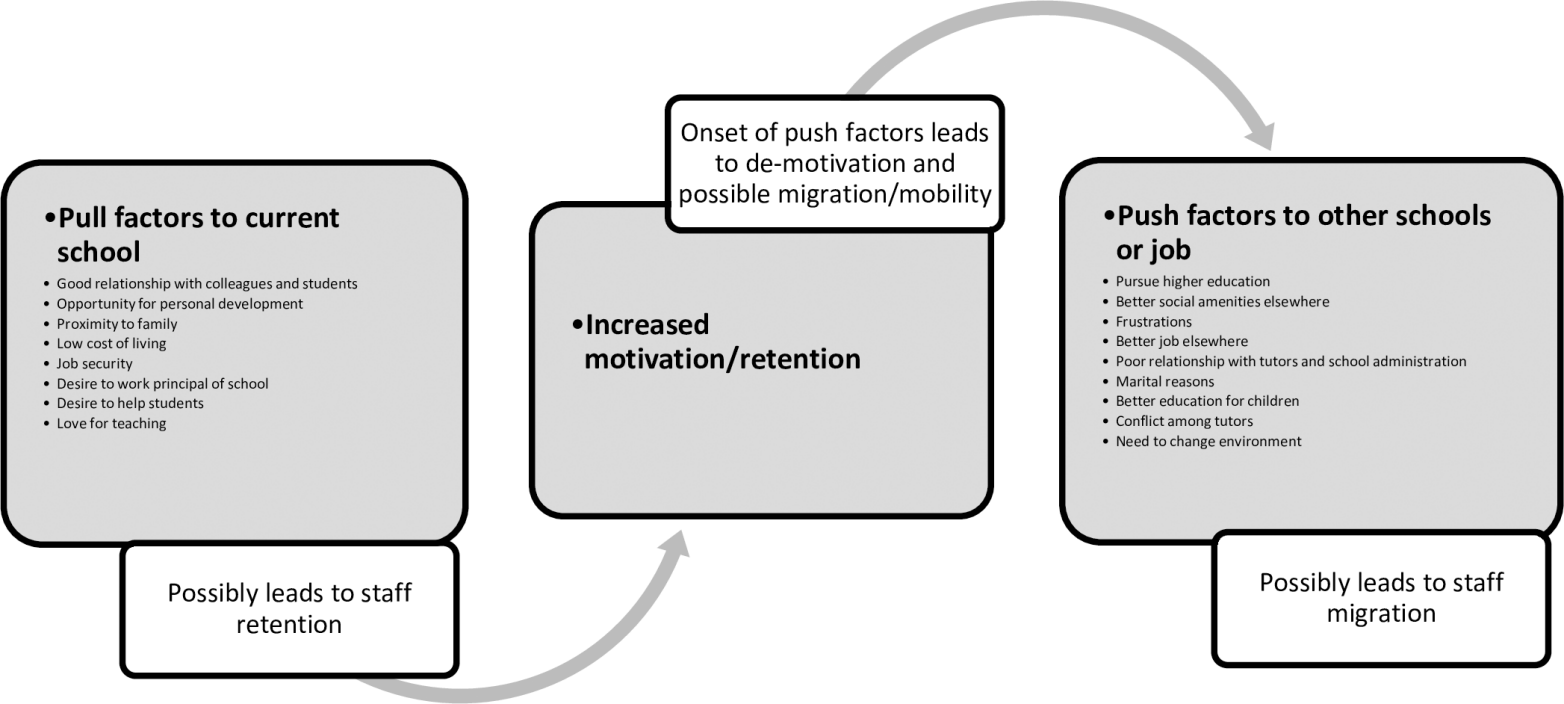
Pull and push factors for health tutors. **Source** Conceptualized by authors (2017).

Work conditions that health tutors mentioned as potential push factors that could compel them to leave their workplaces as health tutors were: the need to change work environment; conflict in school (often among tutors); need to secure better education for children elsewhere; need to join spouse in a different town; poor relationship with colleague tutors or school administration; getting better job opportunity (especially for casual workers); frustrations from the current workplace; unclear role definition for tutors by principals; over-empowerment of principals (described as “tin gods”); lack of delegation of school roles/activities; poor communication among tutors; perceived better working conditions in other training institutions; need to experience and enjoy city life and social amenities (especially among younger tutors in rural schools). Fig 2 shows the sequence of pull and push factors which emerged during the focus group discussions.

### Recommendations towards motivating and retaining health tutors

Participants of the focus group discussions were further asked their opinion on possible interventions towards reducing perceived disparities in health tutor motivation and retention in rural and urban areas. Content analysis of the focus group discussions revealed two main thematic areas, namely health tutor-level recommendations and school administration level recommendations.

At the level of the health tutors, the most recurrent recommendations were implementation of research and book allowances for tutors; introduction of best health tutor award as the case with teachers in the education ministry; introduction of rural allowance for tutors in deprived areas; encouragement of early promotion for tutors in rural deprived areas, and provision of scholarships for deserving health tutors for further studies. A participant in Greater Accra region suggested:

*“…the ministry of health should continue to provide guidelines on promotions and opportunities for further studies and give equal opportunities for tutors to compete for these incentives…”* [Urban FGD Female participant].

A participant in the Northern region made similar submission stating *“…I will suggest health tutors are sponsored to pursue their higher education through exchange programmes besides increasing salaries…”* [Rural FGD Male participant].

Another participant from the Northern region emphasized the need for policy makers to close the widening gap in working conditions of tutors in the rural and urban areas by formulating and implementing policies that will improve working conditions of tutors who agree to work in rural deprived areas.

*“…pay rural allowance to tutors in deprived areas*…*early promotion for those of us [Health tutors] in these [rural] schools…ensure equal opportunities to scholarships and other career development trainings…pay rent allowance to enable us [Health tutors] rent decent accommodation closer to the school…these may help maintain the few tutors here…”* [Rural FGD male participant].

Other recommendations mentioned by participants of the focus group discussions bordered on the need to increase monthly salaries of tutors, and provide uniform or equal fuel and car maintenance allowances. It emerged from the focus group discussions that apparently the amount of money paid as fuel and car maintenance allowances varied from one school to another. While some participants of the focus group discussion bemoaned low fuel and car maintenance allowances, some focus group discussion participants revealed that these allowances were never paid in their schools as mentioned by a participant in Greater Accra region:

*“…like my colleagues mentioned*, *vehicle maintenance allowance and research allowances should be paid for tutors…to motivate us [tutors]…”* [Urban FGD Female participant]

Another participant confirmed opinions of colleague tutors indicating that *“…the Ministry of Health should give policy directives to ensure uniformity in the payment of allowances and other incentives…to ensure fairness…”* [Rural FGD male participant].

Additional recommendations made by members of the focus group discussions were the need to provide policy directive on provision of accommodation for tutors, and institute routine in-service training/orientation for tutors.

*“…the Ministry of Health should consider conducting more trainings and career development pathways for tutors to prepare them for leadership positions…”* [Urban FGD Female participant].

*“…my colleagues mentioned this earlier; but let me add that job descriptions for health tutors need to be updated regularly to meet the increasing demands of health tutors…also routine career development trainings will help keep tutors abreast with the issues…”* [Rural FGD Male participant].

Recommendations made at the level of health training institutions included the provision of regular internet access in the schools, and provision of adequate office space for tutors. A focus group discussion participant in Greater Accra region stated:

*“Offices for health tutors…pay rent allowance to enable us [Health tutors] rent decent accommodation closer to the school…”* [Urban FGD Female participant].

**Fig 3:**
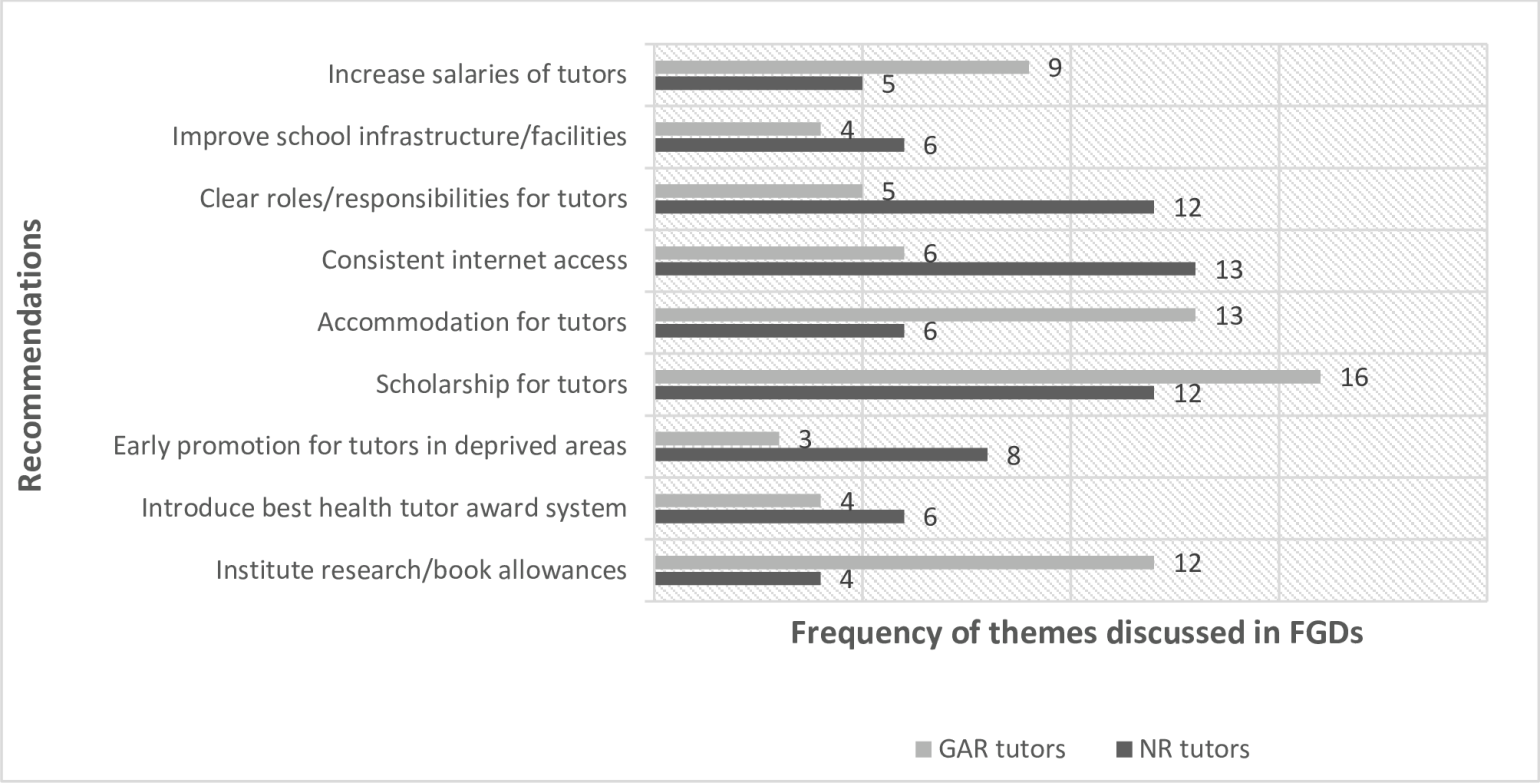
Recommendations to improve staff motivation levels. GAR (Greater Accra Region) NR (Northern Region) FGDs (Focus Group Discussions) **Source** Field Data Greater Accra and Northern Regions (2016).

Other recommendations at the institutional level included: equipping school libraries and resource centres; developing uniform communication and clear role definition for tutors; implementing tenure system for principals; policy providing directive on delegation of duties by principals to tutors; issuing policy directives to schools on uniform school organograms, and investing on school infrastructure. See Fig 3 for details of the most recurrent recommendations mentioned by focus group discussion participants. A participant decried the unavailable of learning material when she said,

*“Teaching and learning equipment are also inadequate…computers…dummies…we don’t even have well equipped skills laboratory for our students…”* [Rural FGD Female participant].

## Discussion

Overall, findings from this study showed that respondents were not content with their work conditions particularly among health tutors in rural areas. These observations appear to corroborate findings of previous similar studies among clinical health workers in Ghana [2–6] and elsewhere [11–15]. Findings of this study on possible factors affecting motivation of health tutors revealed two main thematic areas: health tutor, and school administration. Similar observations were made by Franco et al [16] in their exploratory studies on Jordan and Georgia where individual level determinants and contextual factors were found to influence health worker motivation levels. Even though Franco et al [16] further analysed the direct effect of cultural and environmental differences on motivation levels, this current study emphasized on the rural–urban geographic differences in the perceived working conditions of health tutors.

With regards the health tutors, the most recurrent recommendations made, especially by tutors in rural training institutions, appears to confirm the existing inequities in the working conditions of health workers in Ghana, including health tutors as cited in similar empirical studies on Ghana [5,6,9,17].

This study found out that the amount of money paid as fuel and car maintenance allowances was not uniform; it varied from one school to another. Moreover, while some participants of the focus group discussion complained about low fuel and car maintenance allowances, some tutors said these allowances were never paid in their schools, suggesting lack of uniformity in the payment of these incentives. Lack of uniformity and perceived disparities in worker motivation has been cited as important determinants of staff motivation and migration in the health sector in Ghana [5,9,17].

Provision of staff accommodation and continuous professional development (CPD) have also been found to be key factors that influence staff motivation levels in many sectors including the health sector. In this study, it was observed that many of the health tutors suggested mandatory provision of accommodation for tutors, routine in-service training/orientation for health tutors. In the reviewed literature, a number of scholars advanced similar recommendations towards narrowing existing rural-urban gaps, albeit the focus has long been on clinical health care professionals [17–22]. Even though the Ministry of Health has some existing policies on these work conditions, they have not been effectively implemented over the years due to government budgetary constraints. To promote equity in access to qualified health tutors in Ghana, it is imperative health education policies prioritise rural deprived areas in the allocation and distribution of material resources and creation of equal opportunities for career progression and professional developments. Findings of this current study suggests, many health tutors are compelled to migrate to better endowed schools in the urban areas because they feel cheated and disadvantaged in several respects, comparing themselves to their colleagues in urban areas. According to empirical studies [1–4,22] based on the equity theory of motivation, these perceptions have the tendency of lowering morale and diminishing workers’ output at the workplace.

Previous works on health worker motivation in rural and urban areas in Ghana [5,6,9,17] and other countries [1,22] have alluded to the rural-urban development gaps as important determinants of rural-urban migration of qualified health workers. Although these previous studies did not focus on health tutors, the findings of the current study remain relevant to the ongoing rural-urban migration challenge confronting many health care systems, especially in Ghana and other resource poor countries in the sub-Saharan Africa. As part of measures towards controlling internal and external migration of health professionals including health tutors, national development plans should take into consideration the peculiar development needs of rural communities.

## Limitations

This study relied solely on qualitative data involving 72 health tutors out of the over 1,000 health tutors in the country. Moreover, only two out of the ten (10) regions of Ghana were involved in the current study. In light of this, findings of the study may not necessarily represent the whole country. Nonetheless, the detailed focus group discussions, and varied nature of health training institutions and health tutors enabled the researchers understand the depth of the issues concerning health tutor motivation and constraints which quantitative approaches could not have revealed.

Sampling many more training schools and tutors across the country would help understand the national picture of health tutor motivation, retention and migration patterns in Ghana.

Moreover, due to resource constraints, the current study could not roll out a nation-wide study which could prove beneficial if the Ministry of Health and development partners focus on this important area of the healthcare system. In view of this, future research endeavors could be conducted in larger scales across the over 100 health training institutions in Ghana. Also, employing mixed methods approach in such future studies could help explore in detail work conditions of health tutors and possible associations with the quality of pre-service training being given to health trainees in Ghana.

## Conclusions

The researchers are of the opinion that equitable distribution of health tutors and their retention in rural deprived regions will much depend on equal opportunities in work conditions of health tutors to promote motivation. Promoting equity in work conditions could help control health tutor internal and external migration. Also, supporting health tutors in rural areas to develop themselves in terms of institutionalized continuous professional development (CPD) programmes could help control the increasing migration of health tutors to better endowed urban areas. This approach could help close the existing rural–urban gaps in health tutor motivation in health training institutions in Ghana.

## Declaration

### Ethics approval and consent to participate

Ethical clearance was by the Research Committee of the Health Training Institutions Unit of the Ministry of Health. Moreover, informed consent for the study was obtained from individual health tutors of the sampled health training institutions in line with ethical standards for scientific research. Permission was also sought from the principals of sampled health training institutions before the commencement of the study. Individual participants were assured of their privacy and confidentiality. Responses from the interviews and focus group discussions, as well as the data analysis and reporting was done in anonymity.

For the purposes of ensuring anonymity and confidentiality, true names of the six sampled health training institutions were withheld throughout the paper. Thus, sampled health training institutions in the two study regions were named as follows: *GAR school A; GAR School B; GAR School C; NR School A; NR School B and NR School C*.

## Supporting information

S1 File Qualitative tool for data collection. (DOCX)

## Abbreviations

FGDs: Focus-Group Discussions
GAR: Greater Accra Region
GHS: Ghana Health Service
GHWO: Ghana Health Workers Observatory
HSHR: Health Sector Human Resource
HTSs: Health Training Schools
HTSU: Health Training Institutions Unit
MOH: Ministry of Health
NR: Northern Region
SDGs: Sustainable Development Goals
SSA: Sub-Saharan Africa
WHO: World Health Organization
