# Supplementary material for: Perceived needs of health tutors in rural and urban health training institutions in Ghana: Implications for health sector staff internal migration control
Source: PLoS One. 2017 Oct 5;12(10):e0185748. doi: 10.1371/journal.pone.0185748 (PMC5628878; doi:10.1371/journal.pone.0185748)
Supplement: S1 File — (DOCX) [file pone.0185748.s001.docx]

**S1 File: Qualitative Tool for Data Collection**

**HEALTH TRAINING INSTITUTIONS SECRETARIAT**

**MINISTRY OF HEALTH**

**QUALITATIVE STUDY**

**FOCUSED GROUP DISCUSSION (FGD) GUIDE**

**Topic:** Rural-urban disparities in work conditions of health tutors in Ghana: implications for health sector staff internal migration control

| ***START TIME*** | ***Hr.*** | ***Min.*** | ***Sec.*** |
| --- | --- | --- | --- |
|  |  |  |  |

**PREAMBLE**

This qualitative questionnaire is designed to gather baseline information on factors that influence health tutor motivation and retention and its effects on performance of health training institutions in Ghana.

You are hereby informed that throughout the interview session, your voice will be tape recorded for subsequent transcription and analysis. The information will be used for academic purposes only.

You are also reminded that participation in the personal interview is voluntary and anonymity and confidentiality of personal information is assured. None of your responses here will be directly linked to you in person. You could also decide to withdraw from the interview anytime you so wish to without attracting any consequences. Do you consent to voluntarily participate in this interview? [Individual written informed consent must be signed before commencing the FGDs]. For further information please contact Robert Kaba Alhassan via Tel: (+233(0)241226409) and E-mail ([arkabason@gmail.com](mailto:arkabason@gmail.com)).

**INTRODUCTION**

DATE:……………………………....................................................................................................

NAME OF SCHOOL:……………………………………………………………………………...

NUMBER OF PARTICIPANTS:…………………………………………………………………..

MALES:…………………………………………………………………….....................................

FEMALES:…………………………………………………………………....................................

APROXIMATE OLDEST AGE:…………………………………………………………………..

APPROXIMATE YOUNGEST AGE:……………………………………………………………..

LOWEST EDUCATIONAL QUALIFICATION:…………………………………………………

HIGHEST EDUCATIONAL QUALIFICATION:…………………………....................................

**SECTION A: PRESENT JOB AND PERSPECTIVES ON WORKPLACE MOTIVATION FACTORS**

1. Could you please briefly describe your current jobs/responsibilities indicating your core duties, years of work experience, and reasons for maintaining current job

**NOTE:** Moderator of FGD ensures a brief round table for all FGD members [moderated time 3-5 minutes]

1. Please describe what you think constitutes a well-motivated member of staff and give examples?

**NOTE:** Moderator of FGD ensures participants express their views one after the other giving some examples [moderated time 3-5 minutes]

1. How will you describe your personal motivation levels and that of colleague tutors and why?

**NOTE:** Moderator of FGD ensures each member of the FGD expresses their views without deviating and interruptions from other members [moderated time 3-5 minutes]

1. What factors do you think hinder or promote motivation of staff in this institution and why?

**NOTE:** Moderator of FGD moderates the discussion and ensures each member of the FGD contributes without interruptions [moderated time 2-5 minutes]

1. How will you describe the water and electricity supply situation in this school?

**NOTE:** Moderator of FGD moderates the discussion without interruptions [moderated time 2-3 minutes]

1. How is the transportation situation for staff and students in this institution?

**NOTE:** Moderator of FGD moderates the discussion without interruptions [moderated time 2-3 minutes]

1. How is the logistics situation in terms of modern teaching and learning materials for students and staff?

**NOTE:** Moderator of FGD moderates the discussion without interruptions [moderated time 2-3 minutes]

1. How are the accommodation facilities for staff in this institution?

**NOTE:** Moderator of FGD moderates the discussion without interruptions [moderated time 1-2 minutes]

1. How are the accommodation facilities for students in this institution?

**NOTE:** Moderator of FGD moderates the discussion without interruptions [moderated time 1-2 minutes]

**SECTION B: STAFF AND STUDENT SITUATION IN TRAINING SCHOOLS**

1. How will you describe the staffing situation in this school and why?

**NOTE:** Moderator of FGD moderates the discussion without interruptions [moderated time 3-5 minutes]

1. How do you perceive the workload in this institution?

**NOTE:** Moderator of FGD moderates the discussion without interruptions [moderated time 2-3 minutes]

1. How responsive are the students to staff instructions in this institution?

**NOTE:** Moderator of FGD moderates the discussion without interruptions [moderated time 5 minutes]

1. How are the attitudes of colleague workers in this institution to their other colleagues?

**NOTE:** Moderator of FGD moderates the discussion without interruptions [moderated time 2-3 minutes]

**SECTION C: INSTITUTIONAL AND STAFF PERFORMANCE TARGETS**

1. What institutional targets are set in this school and how are they set?

**NOTE:** Moderator of FGD moderates the discussion without interruptions [moderated time 2-3 minutes]

1. What percentage of your institutional performance target were attained and how were they attained?

**NOTE:** Moderator of FGD moderates the discussion without interruptions [moderated time 2-3 minutes]

1. How will you describe the level of involvement of staff in planning individual and institutional performance targets?

**NOTE:** Moderator of FGD moderates the discussion without interruptions [moderated time 3-5 minutes]

1. How will you describe individual staff performance targets in this school?

**NOTE:** Moderator of FGD moderates the discussion without interruptions [moderated time 3-5 minutes]

1. What percentage of individual performance target were attained and how were they attained?

**NOTE:** Moderator of FGD moderates the discussion without interruptions [moderated time 2-3 minutes]

1. How do you perceive the school’s reputation and recognition from society?

**NOTE:** Moderator of FGD moderates the discussion without interruptions [moderated time 1-2 minutes]

### CLOSING REMARKS

1. What is the most important message that you want us to take away from this discussion?

**NOTE:** Open for all participants to contribute [moderated time 1-2 minutes]

***THANK YOU VERY MUCH FOR YOUR TIME***

| ***END TIME*** | ***Hr.*** | ***Min.*** | ***Sec.*** |
| --- | --- | --- | --- |
|  |  |  |  |
